# Quality of care for patients with non-communicable diseases in the Dedza District, Malawi

**DOI:** 10.4102/phcfm.v7i1.838

**Published:** 2015-06-15

**Authors:** Rachel Wood, Vanessa Viljoen, Lisa Van Der Merwe, Robert Mash

**Affiliations:** 1 Family Medicine and Primary Care, Stellenbosch University, South Africa

## Abstract

**Introduction:**

In Malawi, non-communicable diseases (NCDs) are thought to cause 28% of deaths in adults. The aim of this study was to establish the extent of primary care morbidity related to NCDs, as well as to audit the quality of care, in the primary care setting of Dedza District, central Malawi.

**Methods:**

This study was a baseline audit using clinic registers and a questionnaire survey of senior health workers at 5 clinics, focusing on care for hypertension, diabetes, asthma and epilepsy

**Results:**

A total of 82 581 consultations were recorded, of which 2489 (3.0%) were for the selected NCDs. Only 5 out of 32 structural criteria were met at all 5 clinics and 9 out of 29 process criteria were never performed at any clinic. The only process criteria performed at all five clinics was measurement of blood pressure. The staff's knowledge on NCDs was basic and the main barriers to providing quality care were lack of medication and essential equipment, inadequate knowledge and guidelines, fee-for-service at two clinics, geographic inaccessibility and lack of confidence in the primary health care system by patients.

**Conclusion:**

Primary care morbidity from NCDs is currently low, although other studies suggest a significant burden of disease. This most likely represents a lack of utilisation, recognition, diagnosis and ability to manage patients with NCDs. Quality of care is poor due to a lack of essential resources, guidelines, and training.

## Abstract (fr)

**Abstrait:**

**Qualité des soins des patients atteints de Maladies non transmissibles dans le district de Dedza au Malawi.**

**Introduction:** Au Malawi, les maladies non transmissibles (MNT) sont responsables pour 28% des décès chez les adultes. Le but de cette étude était de déterminer l'ampleur de la morbidité due aux MNT dans les soins primaires, et de vérifier la qualité des soins dans le cadre des soins primaires dans le district de Dedza au centre du Malawi.

**Méthodes:** Cette étude était une première vérification basée sur les registres cliniques et une enquête par questionnaire des personnels de santé qualifiés à 5 cliniques, se concentrant sur l'hypertension, le diabète, l'asthme et l’épilepsie.

**Résultats:** On a enregistré 82 581 consultations, dont 2489 (3.0%) étaient pour les MNT sélectionnées. Seuls cinq des 32 critères structurels ont été remplis aux cinq cliniques et neuf des 29 critères du processus n'ont jamais été mis en œuvre à aucune des cliniques. Les seuls critères de processus accomplis aux cinq cliniques étaient la prise de la tension artérielle. Les connaissances du personnel sur les MNT étaient basiques et l'obstacle principal à la prestation de soins de qualité était le manque de médicaments et d’équipement essentiel, des connaissances et des directives insuffisantes, le paiement pour les services à deux cliniques, l'inaccessibilité géographique et le manque de confiance des patients dans le système de santé primaire.

**Conclusion:** A l'heure actuelle, la morbidité due aux MNT dans les soins primaires est faible, bien que d'autres études suggèrent une charge morbide significative. Cela indique probablement l'absence d'utilisation, de reconnaissance, de diagnostic et de capacité de gérer les patients atteints de MNT. La qualité des soins est mauvaise due au manque de ressources essentielles, de directives et de formation.

## Introduction

According to the United Nations ‘the global burden and threat of non-communicable diseases constitutes one of the major challenges for development in the 21st century, which undermines social and economic development throughout the world, and threatens the achievement of internationally agreed development goals’.^1^ The challenge of non-communicable diseases (NCDs) is seen in Sub-Saharan Africa (SSA), where health systems are already struggling to cope with HIV and AIDS and other infectious diseases, maternal and child health as well as trauma. Most of the premature deaths from NCDs occur in low and middle income countries.^2^ The prevalence of NCDs and their complications have been found to already be high in some SSA settings and may also be underreported.^3^ Studies in SSA show that the prevalence of diabetes mellitus may be as high as 16% in some settings, whilst hypertension ranges from 6% to 48%.^3^ The lack of resources and effective health services for NCDs in many areas leads to late diagnosis and poor management, which impacts on mortality and morbidity.^3^ Epilepsy is also common, with a prevalence of up to 3.7% and is also inadequately managed, with 80% – 90% of patients receiving no treatment. It has been predicted that by 2030, NCD's will cause 46% of all deaths in SSA.

Chronic care for patients with NCDs, such as type 2 diabetes, hypertension, asthma, chronic obstructive pulmonary disease and epilepsy, is usually situated in primary care.^1^ Primary care providers, facilities and services however are often poorly prepared to offer quality chronic care.^1^ High quality primary care for NCDs requires attention to the provision of essential medication and equipment, training of primary care providers, and organisation of the health services to offer care that is patient-centred, timely and efficient, co-ordinated, continuous, comprehensive, integrated and which includes self-management support.^6^

The Malawi government included NCDs in the Malawi Health Sector Strategic Plan for 2011–2016, which indicates their awareness of the issue. According to the plan, ‘the level of hypertension is higher in Malawi (35% of adults) than in the United States of America and the United Kingdom (27%)’. Additionally, it states that NCDs are thought to be a leading cause of deaths in adults, second only to HIV and AIDS. In collaboration with the Ministry of Health, the Malawi Health Sector Strategic Plan focuses on opportunistic screening and treatment of NCDs with cheap, but effective medications.

The motivation for this study came from the healthcare workers at Nkoma Hospital in central Malawi, who wanted to understand more about the morbidity related to NCDs in their hospital and associated primary care platform. A small unpublished survey was performed at the out-patient department and suggested that 40% of patients with NCDs could have been seen at their local primary care clinic, but preferred to travel to the hospital for care. The lack of capacity to care for NCDs in primary care was thought to be placing an additional burden on the hospital and patients. The four main NCDs, as identified by clinicians at Nkhoma Hospital, were hypertension, diabetes mellitus, asthma and epilepsy.

The aim of this study therefore was to establish the extent of primary care morbidity related to NCDs and to audit the quality of care for NCDs (specifically hypertension, diabetes mellitus, asthma and epilepsy) in the primary care setting in the Dedza District of central Malawi. No previous similar studies were identified in the literature from Malawi, and this study should help managers and policy makers to plan better health services for patients.

## Methods

### Study design

This was a baseline audit of primary care morbidity related to NCDs and the quality of primary care. The study included a retrospective survey of the five clinic registers to evaluate the extent of primary care morbidity related to NCDs, and questionnaire-based interviews to obtain qualitative and quantitative data on the quality of care.

### Study setting

Nkhoma Hospital is a mission hospital in the rural Lilongwe region that acts as a referral centre for Lilongwe and Dedza districts. Dedza district was selected as the study setting as another research study was being conducted in the Lilongwe district. Dedza district has 5 clinics: Kalulu, Kasina, Mayani, St. Joseph's and Tsoyo. This platform serves a total population of 98 693. Kasina and St. Joseph's clinics were missionary funded, whilst the others were funded by the government. This results in the need for patients to pay a small fee-for-service at Kasina and St Joseph, but also meant the clinics were slightly better resourced. All of the clinics, except one, had both reliable water and electricity supplies.

None of the clinics had a doctor, whilst in four of the clinics care was provided by a medical assistant, who was a mid-level doctor. At the remaining clinic, care was offered by a nurse. Medical records were patient-retained and only basic information regarding attendance was recorded in the clinic register: date, patient name, age, gender, suspected diagnosis and medications provided.

Prior to the study the hospital had conducted training for the primary care clinics on locally developed protocols for the management of NCDs. The health workers at the hospital had also requested the researchers, who were three medical students attending for a four-week elective period, to conduct this baseline audit of the quality of care on their behalf. The intention was that the results would guide the local health services in how to improve the quality of care in the future.

### Study population

The study included all five of the primary care clinics in the Dedza district, their clinic registers and the most senior clinician involved in NCD care at each clinic.

### Data collection

Data were extracted using a standardised data collection tool from the clinic registers for the previous 14 months (September 2013 to October 2014) on patient consultations for NCDs, their age and gender.

A questionnaire was constructed to obtain information on the structure (e.g. availability of equipment, medication, educational material and human resources) and process of care (e.g. screening, examining, patient counselling and education, management and follow-up). Likert scales were used to evaluate the process of care with a four point scale that ranged from never to every visit. Two sources were used in determining the content of the questionnaire, the integrated audit tool for NCDs developed by the Department of Health in the Western Cape, South Africa¹ and the local NCD clinical management protocols developed by Nkhoma Hospital. The questionnaire also included open questions to document the health workers perspective on the strengths and weaknesses of NCD care.

The interviews with the senior health worker at each clinic enabled the completion of the questionnaire, which was based on their self-reported quality of care. Data could not be extracted from patient records as these were retained by the patients.

### Data analysis

Data was captured on a Microsoft Excel spreadsheet and descriptive statistics were used to determine the frequency of consultations for NCDs out of all consultations conducted at the clinics as well as to determine a mean score or frequency for the criteria used to assess quality of care. The limited qualitative data in response to the open questions was collated and key themes extracted.

### Ethical considerations

Ethical approval for the study was obtained from the Health Research Ethics Committee of Stellenbosch University (S14/08/171) and permission from the local hospital chief medical officer.

## Results

### Primary care morbidity

A total of 82 581 consultations were recorded for the Dedza District primary care platform over the previous 14 months, which included 2489 (3.0%) visits for the designated NCDs. Out of the 2489 visits 891 (35.8%) were for epilepsy, 865 (34.7%) for asthma, 717 (28.8%) for hypertension and 16 (0.6%) were for diabetes. The two clinics (Kasina and St Josephs) run by faith-based organisations had a higher proportion of the visits (1416/2489 [56.9%]) than the three government clinics (1073/2489 [43.1%]).

### Quality of care

This next section presents the results of the audit of clinical care based on interviews with the senior health workers at the clinics.

### Structural criteria

The results of the structural criteria, such as clinical guidelines, basic equipment, self-management resources and medication in stock on the day of the audit are presented in Table 1.

Table 1 gives a clear indication of the limited and often absent structural criteria (protocols, educational material, equipment, medication) at the clinics. None had protocols for any of the NCDs. Most of the clinics had no patient education materials and only one of the five had resources available for every disease.

**TABLE 1:** Results of structural criteria for primary care clinics (*N* = 5).

Structural criteria	*n*
**Diabetes mellitus**	
Protocol on diabetes	0
Scale for weight	1
Height measure	5
Glucometer	2
Blood pressure machine	5
Adult size BP cuff – medium	5
Ault size BP cuff – obese	1
Ophthalmoscope	1
Snellen chart	2
Nylon monofilament	0
Equipment for taking HbA1c blood test	0
Patient education materials	1
Metformin in stock	1
Glibenclamide in stock	1
Insulin in stock	0
**Epilepsy**	
Protocol on epilepsy	0
Patient education materials	1
Phenobarbitone in stock	3
Phenytoin in stock	0
Carbamezapine in stock	1
**Asthma**	
Protocol on asthma	0
Peak expiratory flow meter	0
Patient education materials	2
Salbutamol inhalers	5
Aminophylline tablets	5
Beclomethasone inhalers	1
**Hypertension**	
Protocol on hypertension	0
Urine dipsticks for proteinuria	0
Patient education materials	1
Hydrochlorothiazide	4
Propranolol	1
Atenolol	1

Only the most basic forms of diagnostic and screening equipment were available, in limited supply, or there was a complete absence thereof. All five clinics had a measuring tape and blood pressure machine, but only one had an additional obese size cuff. Only one clinic had a proper functioning weighing scale and only two had a glucometer with which to diagnose diabetes mellitus. No equipment for taking an HbA1c blood test was available in the whole district. There were no peak expiratory flow meters or urine dipsticks.

Regarding medication, only one of the clinics had a supply of oral medication for diabetes and none had insulin. The supply of medication for epilepsy was also limited and no clinics had phenytoin. All five clinics had a supply of reliever asthma medication (salbutamol and aminophyline), but only one had a supply of controller medication (inhaled corticosteroids). Most clinics had access to a thiazide diuretic, but no other medication for hypertension was readily available.

### Process criteria

Table 2 presents the results of the audit on the process of care in terms of the clinic's self-reported frequency of performing key clinical processes.

**TABLE 2:** Results of process criteria for primary care clinics (*N* = 5).

Process criteria	Frequency			
	Never	Once a year	More than once per year	Every visit
**Diabetes mellitus**				
Measure the patient's weight	3	0	1	1
Test the urine for protein	5	0	0	0
Measure the blood glucose	4	0	0	1
Measure the HbA1C	5	0	0	0
Measure the blood pressure	3	0	0	2
Measure the cholesterol	5	0	0	0
Examine the fundi	5	0	0	0
Check the red reflex	5	0	0	0
Test visual acuity	5	0	0	0
Examine the feet	4	0	0	1
Provide patient education	2	1	2	0
**Asthma**				
Counsel about smoking	0	1	0	4
Counsel about inhaler technique	1	2	0	2
Inquiry about number of exacerbations	1	0	0	4
Provide patient education	0	0	2	3
**Hypertension**				
Measure the patient's weight	4	0	0	1
Test the urine for protein	5	0	0	0
Measure the blood glucose	4	0	0	1
Measure the blood pressure	0	0	0	5
Measure the cholesterol	5	0	0	0
Measure the creatinine	5	0	0	0
Counselling on lifestyle changes	0	1	0	4
Provide patient education	0	1	0	4
**Epilepsy**				
Inquiry about frequency of seizures	0	0	1	4
Inquiry about medication side effects	2	1	0	2
Counselling on medication	0	1	0	4
Counselling on lifestyle changes	1	2	1	1
Counselling on alcohol use/abuse	2	0	2	1
Provide patient education	0	1	3	1

Only basic forms of NCD screening and follow-up monitoring were said to be a part of consultations. In most cases, however, these processes did not occur as frequently as optimal standards of care require.

The only process criteria performed at all five clinics was measurement of blood pressure in those with hypertension. Only two clinics stated that they performed any of the process criteria for people with diabetes and only one could actually measure glucose. None of the clinics were able to perform blood tests and even urinalysis or weighing patients was difficult.

Patient counselling and education was variable as some issues were tackled at every visit, such as counselling on medication for people with epilepsy, whilst other issues were less frequently dealt with, such as inhaler technique.

### Qualitative findings

The knowledge on NCD care, with regard to diagnosis and management, of staff members was found to be very basic. This was especially true for diabetes mellitus, where only one interviewee had sufficient knowledge in order to make a diagnosis of the disease.

The main barrier to NCD care was identified as medication shortages. Other prevalent barriers noted were accessibility to the clinics, and affordability of transport costs, fee-for-service and medication at the faith-based organisation's clinics. Other considerations were equipment and staff shortages, lack of training and clinicians’ lack of confidence in their own skills levels.

The barriers acknowledged to play a role in hindering patients’ ability to attend to their medical needs were poverty, lack of NCD knowledge and lack of confidence in the health system. Additionally, health-seeking behaviour was also influenced by patients resorting to care provided by traditional healers.

## Discussion

The quality of primary care for NCDs in the Dedza District of central Malawi is generally poor. In this part of Malawi the commonest NCDs seen in primary care were asthma and epilepsy, and NCDs made up only 3% of primary care visits, in contrast to South Africa, where hypertension and diabetes are the commonest conditions and NCDs make up 14% of visits.^8^

The reported prevalence of NCDs in the region suggest that NCDs are under-represented in Malawian primary care morbidity compared to the expected burden of disease (16% diabetes mellitus; 6% – 48% hypertension and 3.7% epilepsy).^9^The WHO reports that 28% of deaths in Malawi are due to NCDs (cardiovascular diseases 12%, cancers 5%, chronic respiratory diseases 2% and diabetes 1%) and 36.4% of adults have raised blood pressure.^10^ Previous studies have reported that NCDs and their risk factors are major public health problems in Malawi.^10^ The low primary care morbidity from NCDs may therefore represent a lack of ability to diagnose and treat NCDs in primary care or low utilisation of primary care for this purpose. Prevalence rates in rural areas may be lower than in more urban settings, but the findings of this study support the argument that primary care services are unprepared to recognise and manage NCDs.

By international and even regional standards the quality of care in Malawi is sub-optimal, with fundamental gaps in structural criteria such as the availability of basic equipment, supply of medications, implementation of guidelines and capability of primary care providers. The gaps in structural criteria suggest that the resources needed to support the process criteria are largely absent and therefore it is not surprising that the process of care is also reported as poor. It is likely that the actual process of care is worse than the findings of this study as they are self-reported, due to a lack of access to actual patient records. Although patient outcomes could not be measured in this audit they are also likely to be poor.

Many countries in the region are recognising that NCDs are an important part of their burden of disease. For example, Uganda has recognised NCDs as a priority, especially at primary care level, and has developed a department to address research, delivery of interventions and policies.¹¹ This includes the development and testing of standard guidelines for NCD prevention and care. Networks, such as the Chronic Disease Initiative for Africa, have been researching and developing African responses to the growing prevalence of NCDs.¹²

In several countries researchers have been reporting on quality improvement initiatives and demonstrating that significant increases in the quality of care are possible with simple interventions and minimal resource allocation.^13,14^ Interventions that may make an immediate difference to Malawian primary NCD care include:

- Development and implementation of national management guidelines for the commonest NCDs.
- Training and upskilling of primary care providers to diagnose and manage NCDs.
- Supply of essential medication and equipment for management of NCDs at the primary care level.
- Attention should also be given to continuous quality improvement cycles to monitor progress and better informational continuity in the medical record for patients requiring chronic care.

Limitations of this study include the inability to differentiate between new or follow-up visits for patients with NCDs in the clinic registers. It was not possible therefore to determine the actual number of patients with these conditions attending primary care. Data in the clinic registers were sometimes missing, incomplete or illegible. Development of the criteria was strongly based on South African guidelines and audit tools and although evidence-based may not have been entirely appropriate for the Malawian context. Interviews to assess the process criteria were conducted in English, which may have limited the complexity of responses in some cases and were held with the most senior person available at the time of the visit. Clinics were remote and access difficult so that visits had to be undertaken when transport was available.

## Conclusion

Primary care morbidity from NCDs is currently very low in the primary care services of Dedza District in central Malawi, although other studies suggest a significant burden of disease. This most likely represents a lack of utilisation, recognition, diagnosis and ability to manage patients with diabetes, hypertension, asthma or epilepsy. The quality of care is very low with basic structural criteria not met and an inability to deliver on the process criteria. The health system needs to provide guidelines, training, medication and basic equipment to tackle NCDs.

## Appendix 1

### Non-communicable diseases research questionnaire

#### Use of protocols

This refers to the protocols for diabetes, asthma, hypertension and epilepsy provided by Nkhoma Hospital. Training occurred on the 10th of September at Nkhoma Hospital

**Table:**

Question	Yes	No
Are you aware of the protocols?		
Have you read the protocols yourself?		
Have you received training on the use of the protocols?		
Have you tried to implement the protocols		
at your clinic?		

How useful do you think the protocols are for the clinic?

### DIABETES

#### Structural criteria

Which of the following equipment is available and working properly in the clinic TODAY for the management of diabetes?

**Table:**

Question	Yes	No
Protocol on diabetes		
Scale for weight		
Height measure		
Glucometer		
Blood pressure machine		
Adult size BP cuff – medium		
Ault size BP cuff – obese		
Opthalmoscope		
Snellen chart		
Nylon monofilament		
Equipment for taking HbA1c blood test		
Patient education materials		
Metformin		
Glibenclamide		
Insulin		

#### Process criteria

When you treat a patient with diabetes at your facility, how often do you perform any of the following activities?

**Table:**

Question	Never	Once a year	More than one visit per year	Every visit
Measure the patient's weight				
Test the urine for protein				
Measure the blood glucose				
Measure the HbA1C				
Measure the blood pressure				
Measure the cholesterol				
Examine the fundi				
Check the red reflex				
Test visual acuity				
Examine the feet				
Provide patient education				

How would you diagnose a patient with diabetes?

What treatment would you prescribe for a new patient with type 2 diabetes?

### ASTHMA

#### Structural criteria

Which of the following equipment is available and working properly in the clinic TODAY for the management of asthma?

**Table:**

Question	Yes	No
Protocol on asthma		
Peak expiratory flow meter		
Patient education materials		
Salbutamol		
Aminophylline		
Beclomethasone		

#### Process criteria

When you treat a patient with asthma at your facility, how often do you perform any of the following activities?

**Table:**

Question	Never	Once a year	More than one visit per year	Every visit
Counsel about smoking				
Counsel about inhaler technique				
Inquiry about number of exacerbations (assessment of control)				
Provide patient education				

### HYPERTENSION

#### Structural criteria

Which of the following equipment is available and working properly in the clinic TODAY for the management of hypertension?

**Table:**

Question	Yes	No
Protocol on hypertension		
Scale for weight		
Glucometer		
Blood pressure machine		
Adult size BP cuff – medium		
Ault size BP cuff – obese		
Urine dipstix for proteinuria		
Patient education materials		
Hydrochlorothiazide (HTCZ)		
Propanolol		
Atenolol		

#### Process criteria

When you treat a patient with hypertension at your facility, how often do you perform any of the following activities?

**Table:**

Question	Never	Once a year	More than one visit per year	Every visit
Measure the patient's weight				
Test the urine for protein				
Measure the blood glucose				
Measure the blood pressure				
Measure the cholesterol				
Measure the creatinine				
Counselling on lifestyle changes				
Provide patient education				

How useful was the protocol when you tried to use it in the clinic?

How would you diagnose a patient with hypertension?

What treatment would you prescribe for a new patient with hypertension?

### EPILEPSY

#### Structural criteria

Which of the following equipment is available and working properly in the clinic TODAY for the management of epilepsy?

**Table:**

Question	Yes	No
Protocol on epilepsy		
Patient education materials		
Phenobarbitone		
Phenytoin		
Carbamazepine		

#### Process criteria

When you treat a patient with epilepsy at your facility, how often do you perform any of the following activities?

**Table:**

Question	Never	Once a year	More than one visit per year	Every visit
Inquiry about frequency of seizures				
Inquiry about medication side effects				
Counselling on medication				
Counselling on lifestyle changes				
Counselling on alcohol use/abuse				
Provide patient education				

How useful was the protocol when you tried to use it in the clinic?

How would you diagnose a patient with epilepsy?

What treatment would you prescribe for a new patient with epilepsy?

#### Health system performance

What are the main challenges that you experience to offer quality of care for NCDs at this clinic?

What do you think are the main challenges that patient's experience to access quality of care for NCDs at this clinic?

What do you think are the main challenges that patients experience in managing their own NCDs?
